# Comparative regenerative mechanisms across different mammalian tissues

**DOI:** 10.1038/s41536-018-0044-5

**Published:** 2018-02-23

**Authors:** Siiri E. Iismaa, Xenia Kaidonis, Amy M. Nicks, Nikolay Bogush, Kazu Kikuchi, Nawazish Naqvi, Richard P. Harvey, Ahsan Husain, Robert M. Graham

**Affiliations:** 1 Victor Chang Cardiac Research Institute, Sydney, NSW 2010 Australia; 2 University of New South Wales, Kensington, NSW 2033 Australia; 3 Division of Cardiology, Department of Medicine, Emory University, Atlanta, GA 30322 USA

## Abstract

Stimulating regeneration of complex tissues and organs after injury to effect complete structural and functional repair, is an attractive therapeutic option that would revolutionize clinical medicine. Compared to many metazoan phyla that show extraordinary regenerative capacity, which in some instances persists throughout life, regeneration in mammalians, particularly humans, is limited or absent. Here we consider recent insights in the elucidation of molecular mechanisms of regeneration that have come from studies of tissue homeostasis and injury repair in mammalian tissues that span the spectrum from little or no self-renewal, to those showing active cell turnover throughout life. These studies highlight the diversity of factors that constrain regeneration, including immune responses, extracellular matrix composition, age, injury type, physiological adaptation, and angiogenic and neurogenic capacity. Despite these constraints, much progress has been made in elucidating key molecular mechanisms that may provide therapeutic targets for the development of future regenerative therapies, as well as previously unidentified developmental paradigms and windows-of-opportunity for improved regenerative repair.

## Introduction

Regenerative medicine aims to restore tissues, organs, or body parts lost-to-trauma or damaged by disease or aging. Clinically, this represents an enormous challenge because mammals, including humans, display some of the poorest regenerative ability.^1^ A major goal of regeneration research, therefore, is to understand the molecular mechanisms controlling regeneration, since the discovery of a conserved regenerative mechanism could potentially provide attractive therapeutic targets for reactivating latent regenerative responses in adulthood or with aging.

In contrast to mammals, regenerative abilities are robust in many other metazoans, with some taxa of vertebrates (e.g., urodele amphibians) being able to regenerate many different structures throughout life, including entire limbs; a process that generally involves blastema-mediated epimorphic regeneration, as detailed below. It is likely that the ability to regenerate body parts or tissues originated as an epiphenomenon of normal development and growth, which has been selectively lost, rather than evolving de novo as an adaptive trait. To be maintained, an adaptive trait requires selective pressure, but this is lacking, since even in some taxa with robust regenerative abilities repeated predatory loss-of-body parts is not observed. Importantly, related species inhabiting the same geographical region (i.e., sympatric animals) can show contrasting active versus absent regenerative abilities.^2^ Furthermore, while one might reasonably implicate adaptive evolution to explain regenerative responses, such as fin or tail repair in zebrafish (*Danio rerio*) following predatory loss, it is difficult to see how preserved heart or pancreas regeneration in this species could be adaptive, given that predation causing injury to these organs in the adult animal would most likely be lethal. Indeed, as Goss provocatively noted many years ago: “It goes without saying that structures essential to survival cannot regenerate”.^3^

Given that there are examples of complete regeneration of complex structures, which persists into adult life, it would seem fruitful to compare regenerative mechanisms across animal phylogeny, and across taxa showing wide variations in regenerative competence. There are some shared features of regeneration in different animals, such as the processes involved in re-epithelialization, the matrix metalloproteinases involved in degrading or remodeling the extracellular matrix (ECM), upregulation of immune response genes and so on.^4–7^ However, there are also many differences across phyla. For example, regeneration can occur via blastema formation (e.g., regeneration of salamander limbs, tadpole tail regeneration), without proliferation (e.g., morphallaxis in *Hydra*), or by epimorphic regeneration plus morphallaxis (e.g., annelids and planarians).^8^ It also remains unclear if regeneration involves similar molecular mechanisms that are preserved across distantly related taxa, or if the capacity to regenerate damaged tissues is a trait that has evolved repeatedly, albeit by the use of distinct regenerative pathways.

There are several excellent reviews considering studies of regenerative mechanisms across animal phyla.^9–12^ Here, after a brief overview of regeneration types, mechanisms and regulation, we restrict our considerations to regenerative processes used by mammals in tissues that span the spectrum of showing little or no self-renewal (central nervous system, heart), slow cell turnover (liver, pancreas), or active renewal (intestine and skin).^13^ We also consider techniques used to evaluate regeneration, as well as key modulatory factors that are permissive or inhibitory to regenerative responses, such as immunological responses to injury and the ECM.

## Overview of regenerative processes and regulation

### Types and mechanisms of regeneration

Regeneration refers both to the regular and repeated renewal of a particular structure or tissue throughout the life of an organism, that is, the cellular renewal that occurs during normal aging (also called tissue homeostasis or physiological regeneration), as well as to restoration of injured tissue or lost body parts—(also called reparative regeneration). Importantly, the driver of physiological regeneration is replacement to maintain functional homeostasis, whereas reparative regeneration is triggered by injury signals.

A prime example of physiological regeneration in mammals is the seasonal replacement of deer (cervid) antlers.^14^ In most cervids, this is closely linked to androgen levels, being most active prior to rutting when a rise in testosterone causes full mineralization of antler bone. Testosterone levels fall after the rut leading to antler shedding followed by regeneration of new antlers. However, unlike limb regeneration in urodeles, antler regeneration appears not to involve blastema-mediated epimorphic regeneration (see below), but rather an atypical stem-cell-based epimorphic-like regenerative process that is independent of cellular dedifferentiation or transdifferentiation. Other examples of physiological regeneration include replacement of red blood cells, epidermis, endometrium, and gut lining. Homeostatic cell replacement in adult organs involves either stem cell differentiation, or the replication or transdifferentiation of existing cells.^15^

Reparative regeneration can be either incomplete, with only partial restoration of structure and function, or complete, akin to that observed during development. Examples of the former are rare in mammals but include regeneration of digital tips of fetal and juvenile mice, and finger tips of children—a process involving blastema formation that is critically dependent on the nail organ, a keratinized ectodermal appendage unique to the tips of digits.^16–18^ Complete reparative regeneration, as observed with limb regeneration in urodeles, is rarely encountered in mammals, being limited to regeneration after whole-thickness skin injury in certain species of mice (African spiny mice, e.g., *Acomys kempi*) and rabbits (lagomorphs, e.g., *Oryctolagus cuniculus*).^2,12^ This type of injury involves loss of the entire dermis, as well as the underlying connective tissue, blood vessels, nerves, cartilage, and so on, and in *A kempi* all, apart from skeletal muscle, are regenerated, akin to their formation during development.

Types of reparative regeneration include:

(i) Epimorphosis, in which proliferation precedes the development of new tissues. There are two types of epimorphosis:
  - Blastema-mediated epimorphic regeneration. With extreme injury, as occurs with resection of a limb in urodeles or with full-thickness skin injury in mammals, such as mice and rabbits, repair occurs via blastema formation involving locally recruited, lineage restricted progenitor cells that proliferate to form a heterogeneous mass of cells that subsequently undergo maturation, outgrowth and patterning to replace the missing structure.^2^ Hence, new cells generated in this process generally involve proliferation of existing progenitor cells or dedifferentiation of mature tissue, or a combination of both processes.^3,19^
  - Epimorphic or compensatory regeneration. This process results from an apparently precursor/stem cell-independent process that involves the direct recruitment and proliferation of differentiated cells, as observed with liver (see below).
(ii) Morphallaxis. This is observed in invertebrates and occurs through the re-patterning of existing tissue. Importantly, it involves little proliferation/new growth.^20^

Distinct cellular mechanisms that can contribute to mammalian tissue regeneration after injury include:

(i) Differentiation of recruited and/or resident stem and progenitor cell differentiation.^21^
(ii) Replication of differentiated cells. This involves division of existing mature cells (e.g., hepatocytes) and can involve dedifferentiation of existing mature cells, proliferation and re-differentiation, as observed with regeneration of resected zebrafish hearts that results in almost complete structural and functional recovery, and in adult mouse heart following myocardial infarction-induced injury.^22–25^
(iii) Transdifferentiation. This was initially observed for lens regeneration in the adult newt, where pigmented epithelial cells from the iris were found to transdifferentiate into lens cells.^26^ In mammals, regeneration via cellular transdifferentiation is observed in liver and pancreas (see below).

### Regulation of regeneration

Regenerative capacity is regulated by a number of fundamental traits, including age, body size, life-stage, growth pattern, wound healing response and re-epithelialization, ECM dissolution (histolysis), re-innervation, and angiogenesis, as considered in detail for appendage repair.^12^

For example, aging negatively affects regenerative capacity as a result of cellular senescence and telomere shortening; impaired cell differentiation, cell cycle re-entry (dedifferentiation) and cell proliferation; and increased metabolic stress. Aging also impairs re-epithelialization, as is evident from healing by scar formation in older mammals but not their fetal counterparts.^27^ This results in structural changes, such as increased ECM cross-linking, resulting in increased tensile strength and decreased matrix metalloproteinase-mediated histolysis; the latter required to allow cell migration for efficient blastema formation. Increased body size and, hence, increased wound size affect the ability to regenerate by delaying re-epithelialization. An intact nerve supply, by secreting nerve-derived factors, supports regeneration in a wide variety of animals, including *Hydra*, echinoderms, planarians, annelids, and amphibians; fetal wound healing in chickens and mammals; and, as discussed below, regeneration of the injured zebrafish and neonatal murine hearts.^28–31^ Moreover, aging impairs peripheral nerve regeneration in mammals, and in all vertebrates regeneration of nerves is better in younger animals.^11^

The formation of new blood vessels, when there are no pre-existing vessels (vasculogenesis) or from pre-existing vessels (angiogenesis), is required for both tissue homeostasis and for reparative regeneration in adult animals. This is driven by local cues, such as injury-induced wound hypoxia, which results in the elaboration of growth factors e.g., vascular endothelial growth factor and extracellular matrices that are required for blastema-mediated repair.^32^ Additionally, increased age and body size negatively regulate angiogenesis, further reducing the capacity for regeneration. Moreover, even in non-regenerating mammals aging negatively affects the wound healing response to injury.^33^ At the molecular level, differences in regenerative abilities between species have been suggested to result from changes in the genetic and epigenetic circuitry of stem/progenitor cell pools that maintain or restrict access to certain embryonic transcriptional programs.^11,12^

### Techniques to study regeneration

Assessing physiological and reparative regeneration and the mechanisms involved, has been critically dependent on the development of powerful techniques to investigate often minute or very low rates of cell generation and turnover, as well as for the determination of cell cycle activity, transcriptional and post-translational control of gene expression, epigenetic mechanisms, apoptosis, and so on. Application of these techniques may differ depending on cell or tissue context and type of injury. A poor understanding of these techniques and of cell or tissue context may lead to erroneous conclusions.

Table 1 summarizes some of the major techniques developed over the last century that have driven advancement of knowledge in tissue homeostasis and regeneration, and details key strengths and weaknesses.

**Table 1:** Milestones in the development of methods used to study proliferation and regeneration in mammalian tissues

Start date	Strategies and tools	Purpose	Context of initial studies	Pros	Cons
*Equipment and technology*					
~1912	Light microscopy	H&E^1^ staining and light microscopy used to measure the mitotic index of cells	Incidence of mitosis and cell division in the CNS^2^ and in lymphocytes	Specimen preparation is relatively short; can image live cells	Low resolution; specialized training required to identify mitotic phases; scoring cells is time-consuming
~1970’s	Electron microscopy	Assess mitotic figures, cell morphology and tissue structure	Regeneration in intimal thickening, liver, adrenal cortex, and wound healing	High resolution	Specimen preparation and imaging requires specialized training; cannot use live cells; limited field of view
~1970’s	Flow cytometry (FC) and fluorescence- activated cell sorting (FACS)	Rapidly assess cells expressing marker/s of interest and sort accordingly, e.g., cell cycle phase, or live/dead ratios	Sorting T and B lymphocyte populations; identifying cell cycle kinetics in lymphocytes	FC and FACS provide rapid data collection and quantification; increased statistical power with large cell numbers; FACs used to isolate cell populations of interest	Tissue disaggregation may result in cell losses and alter cell morphology; requires the use of well characterized markers; total loss-of-spatial information
~1990’s	Laser scanning confocal microscopy	Spatiotemporal organization of cells in thick optical sections of specimens or isolated cells at high resolution	Cell configuration in the CNS^2^ to elucidate plasticity and function; organization of stress fibers in corneal wound healing	Three-dimensional visualization of specimen using tissue sections or isolated cells	Careful analysis of tissue sections is essential to correctly identify cell types and nuclei; requires the use of well characterized markers
1995	Microarrays	Determine transcripts expressed in tissues or cell populations	First publications of technique	High throughput and rapid analysis of relative expression levels of genes	Genes are selected a priori; the sensitivity range of gene expression levels is lower compared to RNA-Seq^3^
2007 and 2008	RNA-Seq and ChIP-Seq^4^	ChIP-Seq captures DNA bound to histone marks that affect gene expression; RNA-Seq is used to discover the transcriptome of cells or tissue	First publications of technique	Both RNA-Seq and ChIP-Seq are high throughput and large-scale techniques; RNA-Seq provides the most accurate and unbiased method to quantify gene expression	Generates a large amount of data that require bioinformatic expertize
~2014	High throughput single-cell RNA-Seq using microfluidics	Identify the heterogeneous characteristics of individual cells within a cell population in health and disease	Subsets of bone-marrow derived dendritic cells were found to modulate paracrine signaling with other cells	Determines genes expressed at a single-cell level; Drop-Seq^5^ is continually improving to become more accessible	Very expensive and time-consuming to construct many cDNA^6^ libraries (~100–1000 libraries); generates a large amount of data that require bioinformatic expertize
*Cell cycle kinetics*					
1935	Colchicine	Arrests cells in metaphase of mitosis to measure the mitotic index	Characterization of tumor growth and the development of female reproductive organs	Greater chance of capturing cells in mitosis	Colchicine is toxic in high doses; non-specifically targets all cells; scoring mitotic cells is time-consuming
~1960’s	Administration of tritiated ([^3^H])-thymidine	[^3^H]-thymidine is prospectively administered for incorporation during DNA synthesis (S-phase), indicative of cell cycle entry	Labeling DNA synthesis in mouse tissue (intestine, spleen, and pancreas); lineage tracing of osteoclast origins	[^3^H]-thymidine labeling principally identifies cells in S-phase and their [^3^H]-thymidine^+^ progeny	Specimen preparation and processing of autoradiographs is complex and time-consuming, and requires handling radioactive materials; does not signify cell proliferation in cells with incomplete progression through mitosis or cytokinesis; [^3^H]-thymidine labeling is dependent on the method of administration, dosage, and pulse-chase periods
1984	Antibody against Ki67, which is present in G1, S, G2, and M, but not in G0, indicating cell cycling	Marker of cell cycle activity	First publications of technique	Antibody can be used in multiple assays for the rapid assessment of cell cycle activity	Does not signify cell proliferation in cells with incomplete progression through mitosis or cytokinesis
1982	Antibody against BrdU^7^, which is a non-radioactive thymidine analog	BrdU is prospectively administered for incorporation during S-phase, indicative of cell cycle re-entry	First publications of technique	Antibody used in multiple assays for rapid assessment of cell cycle activity; BrdU principally identifies cells in S-phase and their BrdU^+^ progeny; does not require handling of radioactive material	Labels cells in S-phase; does not signify cell proliferation in cells with incomplete progression through mitosis or cytokinesis; BrdU signal is dependent on the method of administration, dosage, and pulse-chase period, which may affect the number of BrdU^+^ cells
~2004	Antibodies against phospho-histone 3 (pHH3) and Aurora B Kinase (AurkB)	Identifies cells in mitosis (pHH3) or cytokinesis (AurkB)	Hepatoblast proliferation in liver morphogenesis; grading cancer cell proliferation in meningiomas	Antibodies used in multiple assays for rapid assessment of cell cycle activity; definitively captures cells in mitosis (pHH3) or cytokinesis (AurkB is present in midbody region)	Labels cells in mitosis or cytokinesis (~80 min), capturing a fraction of cells cycling in this short period of time
2005	Birth-dating	Determine the birth date of cells by correlating ^14^C incorporation levels to atmospheric levels, which sharply increased with nuclear bomb testing and decays over time	Neurogenesis in the human brain	^14^C is quantifiable in human tissue samples	Relies on mathematical algorithms to infer date, and hence, the resolution of cell birth date is ±2 human years
*Genetic toolbox*					
1985	Genetically modified (GM) mice	The mouse genome is altered at the genomic sequence level affecting subsequent expression of the gene of interest	First publications of technique	GM mice represent a mammalian system that can be used to study gene effects in homeostasis and in response to injury and treatment; mice are cheaper to house compared to other mammals	Incomplete characterization of mouse model or inappropriate controls result in misleading conclusions; breeding programs are expensive and time-consuming
1994–1999	Conditional mutant mice (including the Cre-lox system)	Determine cell fate and/or the role of a gene in a spatiotemporal manner	First publications of technique	Allows tissue-specific or ubiquitous gene activation or knockout that is inducible with the simple administration of tamoxifen resulting in Cre recombinase translocation to the nucleus and recombination of genomic loxP sites	Expression of Cre may be ectopic or leaky leading to unwanted off-target effects; inefficient homologous recombination may lead to under-representation of the targeted cell population
2007	Brainbow/Confetti mice	Genetic lineage-tracing of specific cell types and their behavior i.e., clonogenicity	Fate mapping and spatiotemporal distribution of glial and neuronal cells; fate mapping of intestinal Lgr5^+^ stem cells during homeostatic self-renewal	The multiple configurations of fluorescent proteins yield over 90 spectral hues, allowing individual cells to be tagged under the same promoter	Spectral hue configurations are dependent on the transgene and the mouse breeding strategy; factors that alter spectral range, include the promoter fidelity, transgene copy no. and length, and the efficiency and duration of recombination
2013	CRISPR/Cas mediated genome editing in mammals	RNA-guided nuclease system used to rapidly generate GM mice	First publications of technique	Simple, efficient, affordable, and improved transgenesis with the rapid generation of GM mice compared to other genome editing technologies	Off-target mutations

^1^*H&E* hematoxylin & eosin, ^2^*CNS* central nervous system, ^3^*RNA-Seq* RNA sequencing, ^4^*ChIP-Seq* chromatin immunoprecipitation sequencing, ^5^*Drop-Seq* droplet-sequencing, ^6^*cDNA* complementary DNA, ^7^*BrdU* 5-bromo-2′-deoxyuridine

### Role of the ECM in regeneration

The ECM is a complex and dynamic entity that supports and interacts with cells in a tissue to regulate cell proliferation, survival, differentiation, and migration. An understanding of the role of the ECM in homeostasis and disease is, thus, highly relevant to considerations of regeneration. The ECM is produced locally by cells in the matrix and is comprised of fiber-forming proteins (including collagen, elastin, fibronectin, and laminin) interwoven with proteoglycans, in which long-chain negatively charged polysaccharides or glycosaminoglycans form a hydrated gel network in the extracellular space. Other components of the ECM include glycoproteins, ECM-affiliated proteins, ECM regulators, and secreted factors. The ECM is regulated primarily at the post-translational level, with ECM proteins having relatively long half-lives.^34^ Proteomic approaches may thus be more informative for understanding changes in the ECM than genomics approaches.

Biochemical, biophysical, and biomechanical signals between cells and the ECM reciprocally regulate tissue structure and function during physiological and pathological conditions. In the heart after myocardial infarction (MI), for example, robust increases in matrix metalloproteases (MMPs), secreted by inflammatory cells and activated fibroblasts, expose ECM proteins and inflammatory mediators, as well as intracellular proteins, to enzymatic degradation. This, together with the release of a wide variety of cytokines and growth factors, results in ECM remodeling and ultimately organ dysfunction (see reviews^35,36^). Fibrosis occurs when there is a net increase in the rate of synthesis of the ECM. Excessive fibrosis can lead to increased left ventricular (LV) wall stiffness and decreased mechanoelectric coupling, adversely impairing cardiac contractile performance. In contrast, insufficient fibrosis in the heart post-MI can lead to LV wall thinning and rupture. In the liver, hepatic macrophages, by secreting matrix metalloproteinases that can degrade scar tissue, are key cellular mediators of degradation of scar tissue after cessation of injury.^37,38^ An understanding of the molecular mechanisms that regulate ECM-cell interactions may lead to the development of new strategies to enhance regeneration.

#### Effect of matrix and rigidity on cell proliferation

The ECM can affect cell behavior directly, by regulating signal transduction pathways, or indirectly, through changes in local concentrations of growth factors/cytokines/proteins and/or alterations in the physical properties of tissues.^39^ The elasticity of the ECM influences cell shape, cytoskeletal organization, function, protein expression, and differentiation.^40–42^ Elasticity (*E*), determined by atomic force microscopy, varies considerably between tissues. The lateral elasticity of brain matrix is soft (*E*_soft_ ~1 kilopascal, kPa) compared to striated muscle, which is intermediate (*E*_stiff_ ~10–17 kPa) and to osteoid, which is the heavily cross-linked collagen that initiates bone growth (*E*_hard_ ~20–50 kPa).^42^ Following MI, the fibrotic scar that replaces cardiomyocytes (CMs) (*E* ~35–70 kPa) is several-fold stiffer than normal myocardium and mechanically more similar to osteoid.^43^

Stiffness of the ECM can regulate the ability of cells to divide and mature. Murine embryonic fibroblasts, vascular smooth muscle cells, osteoblast and MCF10A mammary epithelial cells proliferate better on a stiff matrix (~24 kPa) than on a soft matrix (~2 kPa).^44^ The mechanical properties of the underlying matrix of CMs have recently been shown to regulate CM proliferation and maturation via effects on the organization of the myoskeleton.^45^ A rigid matrix (2 MPa) facilitates differentiation of cultured rat and mouse CMs, as evidenced by a spread morphology, increased myofibrillar organization, reduced cell cycle activity, and nuclear division (karyokinesis) without cell division (cytokinesis), leading to binucleation.^45^ More compliant matrices (20 kPa more so than 5 kPa) promoted CM dedifferentiation and proliferation, characterized by CM rounding, myofibrillar disassembly, increased CM cell cycle re-entry, cytokinesis, and clonal expansion.^45^ Thus, cytokinesis, but not karyokinesis, in CMs is affected by matrix rigidity. Disruption of the CM myoskeleton with the myosin-II inhibitor, blebbistatin, induces CM cell cycle re-entry, indicating a close association between cytoskeletal organization and cell cycle activity.^45^

The composition of the ECM can affect CM replication. Periostin or agrin increase CM cell counts and BrdU incorporation into DNA in in vitro studies and promotes regeneration after MI, with a decrease in scar volume, and elevation of direct and indirect measures of DNA replication compared to controls, albeit that CM numbers were not determined in these studies.^46,47^ Periostin interacts with integrins, and agrin causes proliferative effects via Yap (yes-associated protein)/ERK signaling pathways. The dystrophin-glycoprotein complex (DGC, a multicomponent transmembrane complex linking the actin cytoskeleton to the ECM) binds Yap to inhibit CM proliferation.^47,48^ The DGC1-Yap interaction is enhanced by Hippo-mediated phosphorylation of Yap after injury to postnatal mouse hearts; Hippo deficiency allows repair without over-proliferation of CMs at the injury site.^48^ The ECM protein, agrin, which is highly expressed in the neonatal heart but wanes with aging, can release Yap from DGC and promote regeneration by CM dedifferentiation and proliferation.^47^ Together, these findings provide strong support for the Hippo pathway and ECM proteins in regulating CM proliferation. It will be of interest to see confirmation of these studies in other mouse models and in larger mammalian models.

The composition of the mammalian ECM also appears to be more inhibitory to cardiac regeneration than zebrafish ECM. Decellularized zebrafish ECM, prepared from normal or healing hearts, was shown to enhance functional recovery and myocardial regeneration when administered into the peri-infarct region of a mouse model of MI, as well as having pro-proliferative and chemotactic effects on human cardiac precursor cells in vitro.^49^ The enhanced regenerative response in the mouse appeared to involve erbB2 signaling as it was abrogated by inhibiting erbB2 activity. Hence, evolutionary differences in ECM appear to influence regenerative capacity.

In the liver, activation of quiescent hepatic stellate cells (pericytes) into activated scar-forming myofibroblasts leads to excessive deposition of ECM, predominantly collagen I and laminin.^50^ Collagen I in the scar inhibits hepatocyte proliferation and must be degraded by MMPs released by hepatic macrophages, before laminin deposition, which is required to facilitate laminin-mediated ductular responses and liver injury repair.^50,51^ Elastin is also expressed from the onset of liver injury. However, in contrast to collagen I, elastin is efficiently degraded in early phases of injury and accumulates only with advanced fibrosis.^37^

In the nervous system, there are three types of ECM: a loose matrix present throughout the brain and spinal cord; matrix resulting from cell membrane-bound molecules; and a unique, lattice-like structure that wraps around specific neurons, called perineuronal nets (PNNs). PNNs are composed of highly negatively charged molecules, including hyaluronan, chondroitin sulfate proteoglycans, link proteins and tenascin R.^52^ Found in the nervous system of a variety of mammalian species, including humans, as well as in birds, such as zebra finch, PNNs limit plasticity in adulthood. This is evident from the finding that their degradation restores the juvenile state, allowing axon sprouting and regeneration of functioning neurons.^53^

New methods that efficiently decellularize tissues and organs in situ will enable high-resolution three-dimensional spatial mapping of ECM architecture in development, homeostasis and disease.^54^ Further understanding of how different ECM architectures affect cell function, proliferation and survival may lead to significant insight into the promotion of tissue regeneration.

### The immune system and regeneration

Regeneration is dependent on an initial inflammatory phase for tissue debridement and protection from invading microbes. However, the immune response must be tightly controlled, both temporally and spatially, as its efficient resolution is critical for successful tissue regeneration. Persistent inflammation leads to poor wound healing, with excessive fibroblast activity, ECM deposition and scarring.^55,56^

The inflammatory cascade varies according to tissue, injury type and regenerative potential.^57^ However, the broad observations after injury are that reactive oxygen species (ROS) are produced, dying cells release damage-associated molecular patterns (DAMPs) and invading microbes release pathogen-associated molecular patterns (PAMPs), which stimulate proliferation and activation of macrophages and recruitment of other immune cells, fibroblasts and mesenchymal stem cells (MSCs).^56^ While a population of monocytes and macrophages are recruited to the damaged tissue, each organ also has its own distinct resident macrophages, which are key to directing the injury response toward repair or fibrosis. After injury in the neonatal heart, for example, embryonic-derived resident cardiac macrophages expand, producing minimal inflammation; whereas, the adult heart contains embryonic-derived resident macrophages that are replaced by proinflammatory monocyte-derived macrophages after injury.^55,58^

Tissue resident macrophages in the mouse originate from one of three lineages, derived from yolk sac, fetal liver or bone marrow, which contributes to their diversity and allows them to play unique roles in organ development, homeostasis, and remodeling.^59^ In the event of an injury, macrophages drive the innate immune response, phagocytosing dead cells and debris and promoting anti-microbial activity via tumor necrosis factor alpha (TNF-α), interleukin 1 (IL-1), and nitric oxide secretion. This initial involvement of macrophages is required for all wound healing, including epimorphic regeneration.^56,60^ As the macrophage population, whether resident or recruited, switches to a pro-regenerative phenotype they produce trophic molecules, such as Wnt ligands to drive cell proliferation and tissue repair.^59^ Resolution of inflammation is essential, as prolongation of the inflammatory phase impairs regeneration and results in fibrosis, which impacts organ function.^56,61,62^ Even in moderately or highly regenerative species, such as anurans and urodeles, respectively, extending the inflammatory phase with pro-inflammatory beryllium sulfate treatment can cause limb regeneration to slow or fail completely.^56^

While an overactive immune response is detrimental to the regenerative process, species with reduced adaptive immune responses—that is, those leading to immunological memory that are activated by antigens and cytokines—have a greater capacity for wound healing and/or regeneration. Compared with the house mouse (*Mus musculus*), gerbils, African spiny mice (more closely related to gerbils than house mice), salamanders, nude mice (*Foxn* deficient), and xid (X-linked immunodeficiency) mice have an enhanced regenerative ability but are deficient in T- and/or B-lymphocytes.^56^ The African spiny mouse, which can regenerate full-thickness 4 mm ear-hole wounds and extensive autotomous dorsal skin loss, has also been shown to have altered innate immunity, with reduced neutrophils and altered macrophage localization and activation compared to *Mus musculus*.^60,63,64^ Some specific strains of *Mus musculus*, including MRL/MpJ, LG/J and LGXSM-6 mice, also have atypical macrophage profiles and are susceptible to autoimmune diseases. These mice are able to heal 2 mm ear-hole punches, albeit without true epimorphic regeneration.^2,60^

In response to antigen and cytokine stimulation, naive T-cells differentiate into T helper (Th) cells and CD4^+^CD25^+^FOXP3^+^ regulatory (Treg) cells. In the skin, Tregs are recruited to full-thickness wounds, where they modulate the inflammatory macrophage response and promote wound closure.^65^ In the liver, Tregs are downregulated after injury to allow for acute inflammation; however, a new Treg population is then recruited to allow for resolution of the inflammatory response to promote effective wound healing.^66^ Tregs promote repair of infarcted mouse hearts by modulating inflammatory responses.^67^ Tregs are also critically involved in zebrafish heart regeneration.^68^ These studies demonstrate that Tregs are important modulators of the immune response across tissues with widely varying wound healing capabilities.

Further supporting the link between immune suppression and regeneration, anti-inflammatories have been used in a number of models to improve regeneration. Mouse skin wound healing is improved and scarring reduced with celecoxib (a COX-2 selective non-steroidal anti-inflammatory agent) treatment.^69^ COX-2 inhibitors have also been used to improve limb regeneration in larval *Xenopus*, whereas the anti-inflammatory thymosin beta4-sulfoxide improves repair of zebrafish and mouse hearts.^70,71^

#### Conclusion

Immune responses are, thus, critically involved in regulating the wound healing process and must be temporally and spatially controlled for epimorphic regeneration to occur. In pro-regenerative species, such as urodeles and the African spiny mouse, it is clear that adaptive immunity and regenerative mechanisms are finely balanced to allow tissue repair. The role of resident and recruited macrophages, and of Tregs, in promoting repair after injury in higher mammals clearly warrants further investigation.

## Tissue-specific regenerative processes

### Regeneration in tissues showing little or no self-renewal

#### Central nervous system (CNS)

The CNS is composed of two major cell types: neurons (electrically excitable cells responsible for transmission of information via electrical and chemical signals), and glial cells, which are divided into oligodendrocytes (responsible for myelination of axons), astrocytes (which interdigitate between neurons and blood vessels), ependymal cells (ciliated simple columnar cells that line the ventricles and central canal of the spinal cord) and microglia (resident macrophages responsible for immune defence in the CNS).

##### Tissue homeostasis

**Fig. 1:**
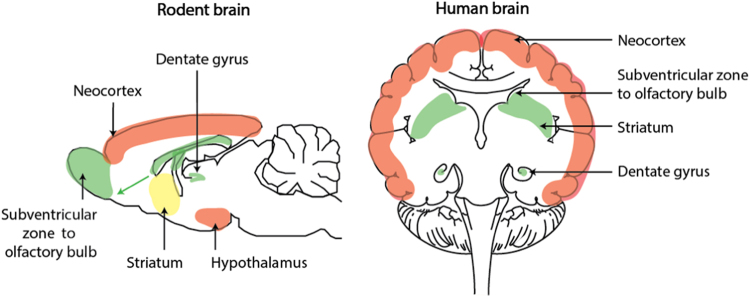
Sites of neurogenesis in the adult rodent and human brain. Regions in which neurogenesis occurs throughout life, in response to injury or regions in which neurogenesis does not occur are indicated in green, yellow, and red, respectively. Figure adapted, with permission from Company of Biologists, from Magnusson and Frisen.^252^

In contrast to most branches of the animal kingdom, which show robust regeneration of their nervous system tissue, the CNS of vertebrates, including humans, has long been considered to be a “stable” or “perennial tissue”, with little or no regenerative ability, the birth of neurons in the mammalian brain having been considered to be restricted to embryonic and early postnatal development.^13^ This view was challenged by a study showing neurogenesis in the adult rodent brain in 1965 (ref. ^72^). Subsequently, adult neurogenesis was documented in the hippocampus of cancer patients, who had been given 5-bromo-2-deoxyuridine (BrdU; a thymidine analog that is incorporated into the DNA of dividing cells that is detected immunohistochemically) for diagnostic purposes.^73^ More recently, studies using retrospective birth-dating based on the integration of atmospheric ^14^C into DNA also showed substantial turnover of adult human hippocampal neurons.^74^ Analysis of ^14^C cell birth-dating indicates that ~35% of hippocampal neurons turn over at a rate of ~1.75% per year, and the rest are static^74^ (Fig. 1). The functional role of these new hippocampal neurons in normal brains, let alone in disease, remains unclear. In mice, hippocampal neurogenesis mediates pattern separation in memory formation and cognition.^75^ Perhaps hippocampal cell turnover subserves a similar function in humans.

There are claims for adult neurogenesis in the neocortex and other brain areas.^76,77^ Despite this, ^14^C cell birth-dating studies provide evidence for the absence of postnatal neurogenesis in all major subdivisions of the human cerebral cortex (estimated turnover of 1 in 1000 neurons every 5 years, as well as in the cerebellum and olfactory bulb (Fig. 1).^74,78,79^ This contrasts with robust adult neurogenesis in the olfactory bulbs of rodents (Fig. 1) and non-human primates.^80,81^ Thus, it appears that neither physiological nor reparative regeneration occurs in human cortical neurons, and there is also no evidence for tissue homeostasis in the olfactory bulb or cerebellum (Fig. 1).

In subcortical regions of the brain adult neurogenesis resulting from proliferation and differentiation of subventricular zone neural stem/progenitor cells (NSCs/NPCs) is observed in the human fetal and infant forebrain, as well as in many other mammals, including rodents, rabbits, and monkeys.^76,82^ In humans the proliferative activity of these cells wanes rapidly after birth, and it remains controversial if neurogenesis continues in this region in the adult human brain.^83^ Neurogenesis, giving rise to new interneurons is observed in adult brains in the striatum, which is adjacent to the subventricular zone.^84^ Striatal interneurons may arise from cells migrating from the subventricular zone, or possibly from transdifferentiation of astrocytes into neurons, in response to ischemic brain injury.^85^

A number of molecular pathways are involved in regulating adult mammalian neurogenesis, including Wnt, Notch, epidermal growth factor (EGF), sonic hedgehog (Shh), bone morphogenetic protein (BMP) and a series of neurotrophic factors.^86,87^ Wnt ligands produced by local astrocytes act via both canonical and non-canonical Wnt signaling pathways to promote the proliferation and neuronal differentiation of subgranular and subventricular zone NPCs.^86–88^ Both Shh and Notch also promote NPC maintenance and proliferation, and suppression of Notch1 correlates with a decrease in neurogenesis with aging.^86,87,89^ One mechanism for Notch1 suppression involves its ubiquitination and degradation, which is mediated by EGF receptor signaling.^86^ BMP, another suppressor of NPC proliferation, promotes cell quiescence, as well as the differentiation of NPCs into glial cells, while supressing a neural fate.^86,87^

##### Response to injury

The finding of mammalian adult neurogenesis has important implications for future regenerative medicine approaches to treating brain injury and disease. NPC proliferation increases in the subventricular and subgranular zones after injury, such as traumatic brain injury or ischemic stroke, resulting in functional improvement; however, this response diminishes with age.^90,91^ In the stroke brain, this expansion of NPCs is facilitated by an upregulation in Notch1 and Shh signaling.^89^ Still, merely regenerating lost cells or damaged axons may not be sufficient. For example, studies of optic nerve regeneration in reptiles indicates that unlike fish, these animals do not recover vision even with axonal regrowth to the tectum, unless they undergo visual conditioning.^92^ In this situation, factors, such as increased brain complexity appear to underlie differences in functional recovery despite similar adequate reparative regeneration.^93^ Following stroke, a period of low-level, spontaneous recovery is observed in both rodents and humans; however, task-specific rehabilitation is required to enhance neuroplasticity and encourage further improvement in disability.^94–96^

Comparative studies of differences in nervous system regeneration in vertebrates also provide important insights into inherent differences in regenerative capacity. Thus, fundamental differences are observed between regenerating species, such as salamanders and larval frogs versus non-regenerating mammals, in the response of radial glial cells, which have proliferative and neurogenic capacity after spinal cord injury.^11^ Only in the former does the response of radial glia, through epithelial to mesenchymal transition, allow reconstitution of the spinal cord neuroepithelial tube that is critical for complete regenerative repair.^97,98^ In mammals, glial cells respond differently, infiltrating the wound and depositing ECM. This leads to glial scar formation, which initially stabilizes the tissue and prevents further damage by necrotic cells, but ultimately impedes axonal extension through the injury, rather than allowing reconstruction of the spinal cord tube or damaged brain.^99–101^ Hence, fundamental differences exist in the properties and responses of glial cells in different animal phyla, which will likely continue to challenge efforts to affect repair clinically in response to nervous system injury.

#### Heart

To maintain high pressure in a closed circulatory system for adequate organ perfusion, the mammalian heart has evolved as a robust contractile organ. This requires adequate embryonic and postnatal development, the latter involving a marked increase in its mass over a relatively brief period (almost fourfold in 25 days between postnatal days 10 (P10) and P25) to adapt rapidly to increases in circulatory demand.^102^

##### Tissue homeostasis

The myocardium is highly vascularised with capillary beds originating from the left and right coronaries, supporting the high demand for coronary blood flow and oxygenation, with a capillary-to-myocyte ratio of 1:1 (ref. ^103^). Of the three major cardiac cell types: CMs, endothelial cells, and fibroblasts, CMs account for 65–85% of the myocardial mass, but only 20–33% of the total cell population of the mammalian heart.^104^ Endothelial cells and fibroblasts are actively renewed throughout life, with predicted annual turnovers of ~17% and ~4% in the adult human heart, respectively.^105^ Lineage tracing studies, ^14^C birth-dating studies, non-radioactive nucleotide incorporation studies, and stereological CM counting studies indicate that CM turnover is detectable in the young but declines rapidly with age (~0.76% per year in mouse; 1% per year at age 25 years falling to 0.45% per year at age 75 years in humans).^105–108^ In the mouse, CM generation has been reported to be restricted to a small fraction (<0.2%) of mononucleated CMs.^106^ However, this study could not rule out contributions from binuclear CMs.

It is now generally agreed that postnatal CM generation is due to proliferation of existing CMs, rather than to maturation of stem/precursor cells.^106,109^ The number of CMs increases postnatally in the rat and mouse.^102,110^ Continued postnatal CM generation is consistent with a greater increase in heart weight than in body weight in the period immediately before adolescence, which is due to a surge in circulating thyroid hormone (T3) levels as the hypothalamic/pituitary/thyroid axis matures and also due, in part, to hypertrophic CM growth during this time.^102^ An increase in CM population number was also observed in humans during the first 20 years of life, from 1 billion at birth to 4 billion in adults.^107^ Design-based stereology to quantitate CM numbers showed that CM generation in human hearts is robust in early childhood but then declines, so that of the ~40% of CMs generated throughout life, only 3% are “born” after age 10 years.^105^ Surprisingly, CM numbers remained constant throughout life (3.1 billion CMs at birth and in adults), implying that the robust CM generation observed during infancy is not due to continued postnatal growth of the heart but, rather, to replacement of lost CMs, although cell death was not evaluated in this study.^105^ Another study, however, using the same donor hearts found no evidence of apoptosis during this time.^107^ Assuming that most of the CM generation observed postnatally in human and mouse hearts is involved in cardiac growth, then that involved specifically in tissue homeostasis, particularly during adulthood, must be exceedingly small, i.e., only a fraction of the ~0.76% turnover per year in mouse and the 0.45–1.0% in human hearts; a conclusion consistent with the finding that contrary to popular belief, CM apoptosis is minimal throughout life and does not increase with aging in the normal human heart.^111^

Maturation of the hypothalamic/pituitary/thyroid axis occurs earlier (toward the end of pregnancy) in humans than in rodents.^112^ However, the finding that continued hyperplastic CM growth in preadolescent mice is T3-dependent is likely of clinical significance, since Bernhard Kühn et al. (personal communication) have shown that in contrast to healthy children, CM generation in infancy is suppressed in infants with congenital heart disease; a condition known to have impaired T3 production.^112^ Moreover, thyroid hormone production is exquisitely regulated by nutritional status, which may negatively impact heart development not only in children with congenital heart disease, but also in the 165 million globally, who are stunted from malnutrition.^113^ This effect may potentially impair their response to, and survival from, myocardial injury later in life.

##### Response to injury

Unlike zebrafish hearts, adult mammalian hearts are unable to regenerate cardiac tissue following injury, which ultimately leads to heart failure. In the US, over 715,000 people/year suffer from MI. If complete cardiac regeneration could be achieved, this would lead to marked improvements in quality of life and decreases in healthcare costs.

Repair of the embryonic mouse heart following ablation of 50–60% of cardiac progenitor cells or immature CMs is complete with full regeneration.^114^ Histologically, the myocardium regenerates with resolution of scar/clot and increased DNA proliferation, and function of the intact heart is restored. New CM proliferation involves an increase in the proliferation rate of immature CMs, above the already brisk proliferation rate observed at this time in uninjured hearts.^114^ This level of replacement is almost on par with that observed in zebrafish, where 70–80% of lost CMs can be replenished.^115^

Complete cardiac regeneration, evident by complete histological repair and clot resolution, is observed in neonatal mice following resection of the LV apex, MI due to occlusion of the left anterior descending coronary artery, cryoinjury or genetic CM ablation (see review^109^). Additionally, total CM numbers increased with cardiac function returning to baseline. Regeneration following apical resection or MI is limited to a brief developmental window, being robust when effected in P1 hearts but not P7 hearts; a time when CMs have exited the cell cycle and are quiescent.^109^ However, consistent with CM re-entry into the cell cycle during preadolescence, myocardial ischemic (MI) injury at this age leads to a partial regenerative response. This is evident by decreased scar size, increased BrdU labeling and improved ejection fraction compared to mice experiencing MI injury at a later age (P21).^102^ In the neonatal mouse infarct model, regeneration has been shown to involve not only CM replication but also robust angiogenesis and revascularisation.^109^

Cardiac regeneration with full functional recovery has been reported in several case studies of infants and children afflicted with diphtheria or after a perinatal infarct.^116,117^ Scarless repair of the myocardium has also been observed after corrective surgery for a congenital cardiac anomaly.^118^ Thus, although definitive evidence of CM regeneration could not be obtained in these studies of human hearts, it appears that in infancy, humans, like other mammals, can repair their hearts in response to injury, with apparent complete structural and functional regeneration of the myocardium.

CM renewal is driven by division of pre-existing CMs. Initially it was suggested that only mononuclear CMs are capable of dividing. However, mature, preadolescent binuclear CMs can re-enter the cell cycle, replicate and contribute substantially to postnatal growth of the heart.^102^ This finding has been confirmed in adult CMs.^25^ Repair of the heart following injury has been suggested to involve CM dedifferentiation, division and re-differentiation.^24,25^ Dedifferentiation is characterized by disassembly of sarcomere structure, extrusion of mitochondria, electrical uncoupling, and expression of precursor cell markers and of regulators of cell cycle progression.^24,47,119^ Re-differentiation involves restoration of cell morphology, sarcomere organization and contractile function.^120^ Adult CMs subjected to ischemia undergo dedifferentiation, proliferation and re-differentiation as evidenced in an in vitro co-culture model (adult CMs co-cultured with neonatal rat ventricular myocytes), as well as in post-infarct hearts.^25^ Ischemia induces gap junction uncoupling in the peri-infarct zone as a result of hypoxia-mediated dephosphorylation of the gap junction protein, connexin 43—the major mediator of intercellular communication, including propagation of calcium transients. Such uncoupling is thought to reduce the spread of proarrhythmic membrane depolarization signals from dying CMs to surviving myocardium.^121^ This uncoupling may be important for dedifferentiation of ischemic CMs, but may also impair CM re-differentiation since adenoviral expression of an ischemia-resistant connexin 43 mutant 3 days post-infarct enhanced re-differentiation and improved cardiac dimensions and function measured 6 weeks after infarction.^25^ While these improvements in structure and function were marginal, they were statistically significant. Cardiac electrical activity was not evaluated in this study, so it is possible that beneficial re-differentiation may be offset by an increased risk of arrhythmias. Nevertheless, approaches that could safely enhance this process could be a significant step in regenerating myocytes that would otherwise die or be unable to participate in the regenerative process following an ischemic injury.

A variety of factors have been implicated in regulating the mammalian CM cell cycle, including micro RNAs (miRNAs), cyclins, transcription factors and the DNA damage response, and efforts to alter these pathways have resulted in enhancement of regeneration, replication or survival in adult hearts (see review^122^). For example, Hippo signaling is an evolutionarily conserved kinase cascade that regulates a variety of cellular functions, including proliferation, survival, differentiation and organ size.^123^ Overexpression of a constitutively active form of its effector, Yap1 in the adult mouse heart induces partial cardiac regeneration and improves contractility after MI.^124^ Overexpression of wild type Yap1 stimulates proliferation of postnatal CMs.^125^ Moreover, CM-restricted loss-of-YAP1 causes lethal hypoplasia and restricts neonatal heart regeneration.^125^ Thus, the Hippo/Yap1 signaling pathway appears to be a critical regulator of CM proliferation, via a pathway involving PI3K/AKT and the subunit *Pik3cb*.^126^ The transcription factor, Meis1, is also a potential target for re-activating CM proliferation, with Meis1 silencing in P1 mice resulting in a profound increase in CM proliferation without evidence of hypertrophy or cardiac dysfunction, and Meis1 overexpression in P1 mice inhibiting the regenerative response following ischemia/reperfusion.^127^ Infarct studies have not yet been done on adult mice, hence further studies are required to better evaluate the benefit of Meis1 manipulation for cardiac regeneration.

One of the most dramatic physiological changes that occur at birth is the change from the hypoxic environment of the fetus to the oxygen-rich environment of the postnatal organism. Increasing levels of mitochondrial ROS and hydrogen peroxide (H_2_O_2_) in early postnatal murine CMs have been reported to activate the DNA damage response pathway and cause cell cycle exit.^128^ However, daily N-acetyl cysteine (NAC) injection for 21 days from P1, to scavenge ROS during the transition from anaerobic to aerobic metabolism, had no effect on CM numbers vs controls at P7, a time point by which ROS is proposed to have caused cell cycle arrest.^128^ There was, however, an increase in CM number in the NAC-treated group by P14. NAC can be an oxidant (H_2_O_2_ producer), and have anti-apoptotic and pro-proliferative effects, independent of its antioxidant (ROS scavenging) activity.^129–132^ This makes interpretation of results obtained with NAC difficult.

Exposure of normal or infarcted adult mice to severe hypoxia has been reported to result in a decrease in ROS production and oxidative damage, and an increase in heart growth due to CM hyperplasia, as well as to decreased scar formation in infarcted mice.^133^ However, given that hypoxia is a known proliferative factor in the heart that promotes angiogenesis and cell expansion, it is difficult to distinguish the effect of ROS reduction versus hypoxia as the driving force for proliferation. In contrast, transgenic overexpression of the H_2_O_2_-generating enzyme, NADPH oxidase 4 (Nox4), resulted in increased ROS synthesis, higher CM number, elevation of cell cycle activator cyclin D2 and increased cardiac mass at 1–3 weeks of age.^134^ In addition, H_2_O_2_ promotes heart regeneration in zebrafish independent of immune cell recruitment.^135^ Thyroid hormone is known to increase aerobic metabolism, induce mitochondrial biogenesis and activate oxidative phosphorylation (OXPHOS), a major source of ROS, in the early postnatal period.^112^ Thyroid hormone has been reported to induce expansion of CM numbers in the preadolescent heart.^102^ This suggests that H_2_O_2_-ROS derived from mitochondrial OXPHOS may promote CM proliferation.

### Regeneration in tissues showing slow cell turnover

#### Liver

**Fig. 2:**
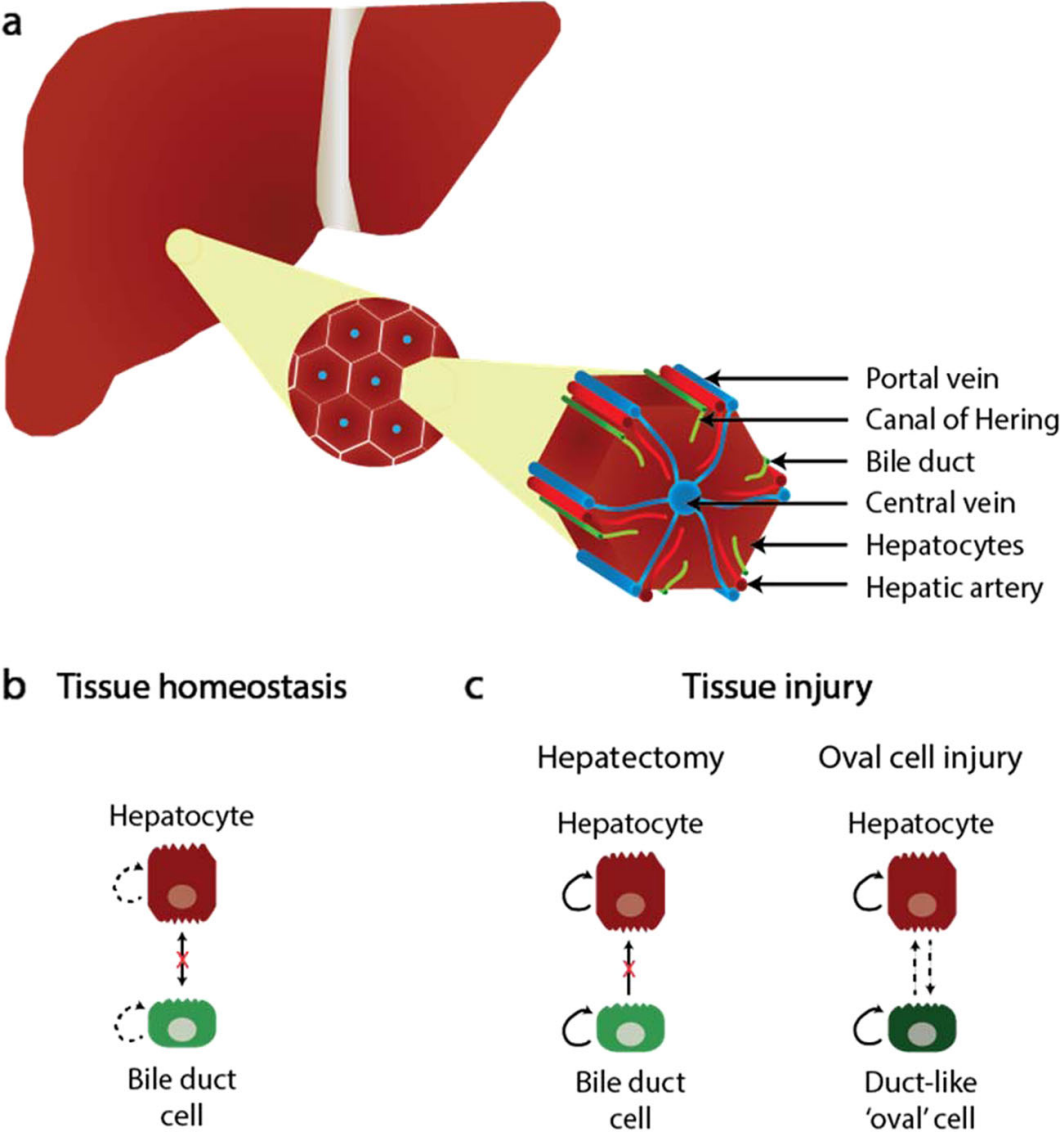
Architecture of the adult liver. **a** Hepatocytes are perfused by blood from the portal vein and hepatic artery, which flows into the central vein. Bile, secreted by hepatocytes, is transported through the canal of Hering to the bile duct. **b** Tissue homeostasis involves limited self-renewal (dashed arrows) of hepatocytes and bile duct cells, with no interconversion between these cell types. **c** After hepatectomy, both bile duct cells and hepatocytes can self-renew, but bile duct cells do not become hepatocytes. In the oval cell response, adult hepatocytes and periportal ductal ‘oval’ cells in the canal of Hering proliferate; oval cells differentiate into hepatocytes to replenish hepatocyte numbers when hepatocyte replication is impaired. Figure adapted, with permission from Springer Nature, from Kopp et al.^15^

The liver is connected to the systemic circulation via the hepatic artery and portal vein. These subdivide into small capillaries known as the liver sinusoids that lead to the lobules—the functional units of the liver. The liver consists of two main epithelial cell types: (a) hepatocytes, which perform metabolic activities, including bile secretion and (b) bile duct epithelial cells (cholangiocytes), which form conduits for the transport of bile to the intestine (Fig. 2).

##### Tissue homeostasis

Polyploidy is a characteristic feature of mammalian hepatocytes, being 20–40% in the adult human liver, 80–90% in adult C57BL/6 mice and 70–80% in adult rats (see reviews^136^). Hepatocyte polyploidization starts at the suckling-weaning transition (3 weeks postnatal) in rodents, with the generation of binucleated tetraploid (2 × 2*n*) or mononucleated tetraploid (4*n*) hepatocytes.^137^ Polyploidization results from a failure of cytokinesis, which is attributed to a combination of increased insulin-PI3K/Akt signaling, increased E2F8 or decreased E2F1 transcription factor expression, and to increased miR-122 expression, inhibiting the expression of pro-cytokinesis factors.^136^ Octaploid (binucleated 2 × 4*n* and mononuclear 8*n*) hepatocytes accumulate by 2–3 months postnatally.^136^ Hepatocyte polyploidization increases during normal aging, as well as during repair in response to surgical resection, toxin injury, metabolic overload, or oxidative damage (see below).

In adults, epithelial cell turnover is reported to be very slow (<0.005% of hepatocytes are mitotic at any given time).^15^ In contrast to the intestinal crypt, which is replaced every 5 days and the skin, which is replaced every 12–30 days (see below), the normal liver is estimated to be replaced approximately once a year.^15^ There is no evidence for the contribution of a non-hepatocyte stem cell^138^ (Fig. 2). Expression of the Wnt target gene, Lgr5 (leucine-rich-repeat-containing-G-protein-coupled receptor 5, an agonist of the Wnt pathway known to be a marker of several epithelial stem cell populations), is low in the liver during homeostasis.^139^ Recent work suggests that hepatocytes are divided into two distinct populations based on their metabolic zonation: pericentral and periportal hepatocytes. Wnt signals from central vein endothelial cells maintain a population of Lgr5^+^-Axin2^+^ pericentral hepatocytes (located adjacent to the central vein in the liver lobule).^139,140^ These cells have been reported to be responsible for populating the entire lobule during homeostasis.^140^ This does not occur during regeneration (see below). Consistent with this notion, independent work shows that during liver homeostasis there is a reduction in the number of periportal hepatocytes expressing the periportal zonation marker, Mfsd2a (major facilitator super family domain containing 2a).^141^ However, other work shows that Lgr5^+^-Axin2^+^ pericentral hepatocytes rarely proliferate and that Lgr4^+^ parenchymal hepatocytes account for most new hepatocytes during liver homeostasis.^139^

##### Response to injury

The liver has been known since antiquity for its ability to completely restore its mass and function after injury; however, chronic injury leads to scarring.

The liver uses two distinct mechanisms for repair depending on the mode of injury: proliferation of stem/progenitor cells (oval cells) following exposure to toxins or viruses, or replication of existing hepatocytes following surgical removal of parts of the liver (hepatectomy). The oval cell response is an intense proliferation of periportal ductal cells (oval cells containing oval nuclei with a high nuclear-to-cytoplasmic ratio) in the canal of Hering.^142^ Oval cells have long been considered facultative bipotential liver stem/progenitor cells. In vivo lineage tracing studies have shown that in prototypical mouse models of oval-cell activation, adult hepatocytes are generated by self-duplication and rarely from oval cells.^143–145^ However, recent in vivo lineage tracing work indicates that if hepatocyte replication is impaired in ductular reaction models, thereby recapitulating human disease, physiologically significant regeneration can result from cholangiocyte-derived hepatocytes^146^ (Fig. 2c).

Hepatectomy has been studied in adults since 1931 (ref. ^147^) and mechanisms of repair after hepatectomy have been extensively reviewed.^148^ Liver mass is restored within 7–10 days in rodents and after 6–8 weeks in humans.^149,150^ However, unlike regeneration of the resected liver in zebrafish, which undergoes epimorphic regeneration, the normal architecture of resected adult mammalian liver lobes is not regenerated in this way. Rather, repair after hepatectomy involving up to 30% of the liver mass, in the adult rodent, is achieved by hypertrophy of the remaining hepatocytes in all residual lobes, resulting in increased metabolic activity without hepatocyte division. In this form of repair, the proportion of binucleated hepatocytes remains unchanged.^151^ Surgical resection of up to 70% of the adult liver results in hepatocyte hypertrophy followed by cell proliferation. Here, the proportion of binucleated hepatocytes decreases, suggesting that binuclear hepatocytes enter M phase and undergo cytokinesis to produce two mononucleated daughter cells.^151^ As in the heart, lineage tracing studies of hepatocyte proliferation in both rats and mice indicate that stem cells are not involved in restoring adult liver mass^138^ (Fig. 2c). After injury in the adult rodent and human liver, including oval-cell inducing injuries, pericentral Lgr5^+^ cells, which are capable of clonogenic growth, appear near bile ducts.^152,153^ Although this coincides with robust activation of Wnt signaling, these cells do not contribute substantially to regeneration post hepatectomy.^139^ They do not spontaneously differentiate into hepatocytes in vitro but do so with low efficiency upon transplantation.^152,153^ Thus, Lrg5^+^ cells are not considered bona fide bipotential hepatic stem cells.^15^ Rather, recent work indicates that Lgr4, which is expressed in virtually all hepatocytes across the liver lobule, has an important dominant role over Lgr5 in Wnt/β-catenin signaling and hepatocyte proliferation during liver regeneration.^139^ Other work has reported that in the early phases of liver repair following 70% hepatectomy, Mfsd2a^+^ periportal hepatocytes undergo proliferation to replace the pericentral hepatocyte population throughout the whole liver.^141^ After injury recovery, the Mfsd2a^+^-derived hepatocytes are then reprogrammed into pericentral hepatocytes.^141^ The repair process appears to be coordinated by liver sinusoidal endothelial cells (LSECs), which activate vascular endothelial growth factor receptor 2 (VEGFR-2) and tyrosine kinase with immunoglobulin-like and EGF-like domains 2 (TIE-2) signaling, resulting in the secretion of angiocrine factors (Wnt2 and hepatocyte growth factor), and cytokines (CXCR4 and CXCR7) that trigger hepatocyte proliferation and liver repair.^154,155^ Hepatic stellate cells (liver pericytes) are also activated to secrete hepatocyte growth factor and hedgehog, which stimulate hepatocyte proliferation.^148^ In addition, hepatic macrophages upregulate Wnt signaling in response to phagocytosis of dead cells.^156^

In contrast to the adult liver, 20–30% hepatectomy in neonatal (day 0.5) mice results in numerous rounds of clonal cell division and full reconstitution of lobe architecture.^157^ This is similar to the regeneration observed in the neonatal heart.^158^

Liver repair following 70% hepatectomy is associated with dynamic changes in the expression of specific miRNAs, such as miR-21, miR-221, and miR-26a (refs. ^159–161^). These miR changes correlate with changes in the expression of target genes that play important roles in liver repair, such as those encoding growth factors or cell cycle regulators. Recent studies also indicate a role for long non-coding RNAs (lncRNAs) in liver repair, examples being LALR1 (lncRNA associated with liver regeneration 1) and MALAT1 (metastasis-associated lung adenocarcinoma transcript 1).^162,163^ These lncRNAs promote cell cycle progression and accelerate hepatocyte proliferation by activating Wnt/β-catenin signaling.

In chronic liver injury, such as that induced by chronic CCl_4_ administration, pericentral hepatocytes die and are replaced by Mfsd2a^+^ periportal hepatocytes that are gradually reprogrammed into pericentral hepatocytes, as was seen with 70% hepatectomy.^141^ However, the profibrotic CXCR4 pathway is activated in LSECs in this type of injury, with CXCR7 signaling reduced. This results in the proliferation and activation of hepatic stellate cells into myofibroblasts, which leads to liver fibrosis and the inhibition of hepatocyte proliferation.^148,155^

Recent experiments have provided evidence that human and mouse hepatocytes can reversibly transdifferentiate into ductal biliary epithelial cells, which expand and subsequently contribute to restoration of hepatocyte mass by re-differentiation into functional hepatocytes.^164^ These hepatocyte-derived duct-like cells thus display the properties previously ascribed to classic oval cells: they are marked by induction of mesenchymal markers, stem/progenitor markers, and signaling pathways that activate and maintain endothelial-to-mesenchymal transition, including the Wnt/β- catenin, TGF-β, Notch, and hedgehog pathways. Results are consistent with the Hippo signaling pathway maintaining the differentiated hepatocyte phenotype, whereby a loss-of-Hippo signaling leads to activation of the transcriptional coactivator, YAP, and downstream Notch signaling, resulting in hepatocyte dedifferentiation to oval cells and conversion into biliary cells.^165^

##### Conclusion

Based on current evidence, the plasticity of differentiated cells contributes to tissue repair in the liver under both homeostatic and injury conditions, with cholangiocytes acting as facultative liver stem cells to effect repair when hepatocyte regeneration is impaired.

#### Pancreas

**Fig. 3:**
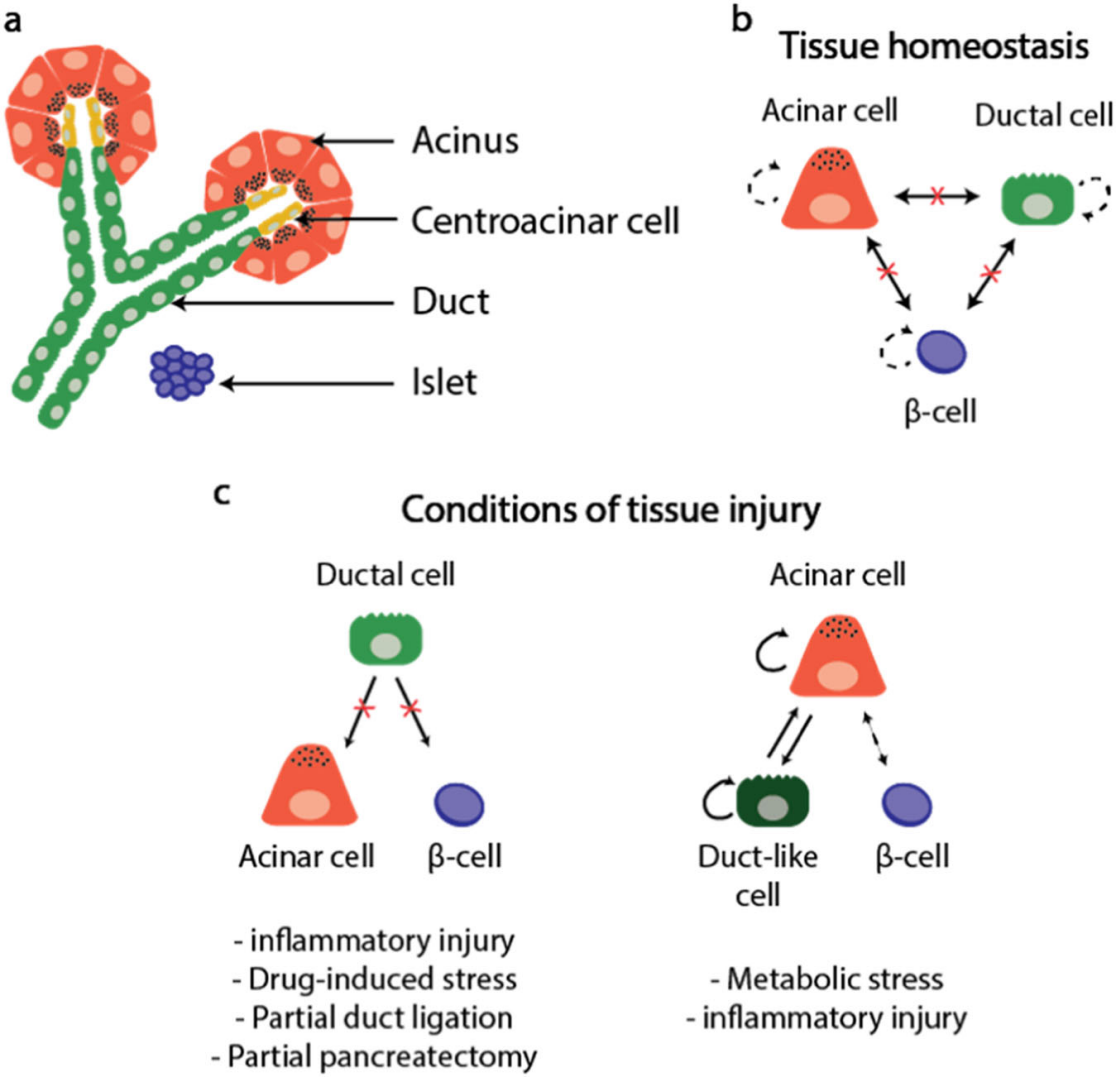
Architecture of the pancreas. **a** Functional units of the adult pancreas are made up of acinar, centroacinar, and ductal cells and are interspersed with islets of endocrine cells (β-cells). **b** During tissue homeostasis, acinar, ductal and β-cells are capable of some self-renewal (dashed arrows), but there is no transdifferentiation between the cell types. **c** Cell responses to injury depend on the injury type. Clonogenic ductal cells are unable to convert onto acinar cells or β-cells. Acinar cells convert to duct-like cells, which then return to an acinar cell phenotype. Figure adapted, with permission from Springer Nature, from Kopp et al.^15^

The pancreas is comprising two functional components: (a) exocrine tissue, which includes digestive enzyme-secreting acinar cells arranged in functional units called acini, and ductal cells that form the conduits responsible for passage of these enzymes into the gut; and (b) endocrine tissue, which includes islets of Langerhans containing insulin-producing β-cells, glucagon-producing α-cells, somatostatin-producing δ-cells, ghrelin-producing ε−cells and pancreatic polypeptide-producing γ-cells—each secreting their hormones into the circulation where they play a major role in regulating glucose metabolism (Fig. 3).

##### Tissue homeostasis

In adults, acinar, ductal, and endocrine islet cell types are long-lived (estimated to survive more than a year in mice) and have low-proliferation rates (estimated at <0.2% per day).^15,166,167^

Physiologically, β-cell proliferation in humans and rodents occurs at a low level (1–3% of human cells and 10–30% of murine cells in cell cycle) in neonates and the early stages of life, after which there is an age-dependent decline in β-cell proliferation (to ~0.1–0.2%).^15,167^ During postnatal life, β-cells and acinar cells are produced by the division of pre-existing cells^168–170^ (Fig. 3b). Mitogenic signaling pathways have been studied extensively in adult rodent β-cells; however, adult human β-cells fail to respond to the same growth factors and nutrients.^171^ For example, human β-cells do not proliferate during pregnancy or in response to insulin resistance.^172^ It remains unclear why mitogenic pathways are not activated in human β-cells.

In adult human β-cells, most of the key G1/S molecules, such as cyclins and cyclin-dependent kinasese (CDKs), are not found in the nucleus, but in the cytoplasm.^173^ Thus, replicative quiescence in β-cells might be due in part to the inability of cyclins and CDKs to access the nuclear compartment and this may be a common feature of other non-proliferating adult cells, such as skeletal muscle, CD8^+^ memory T cells, keratinocytes, and prostate gland cells.^171^

miRNAs regulate β-cell proliferation. One example is miR-7a, which inhibits adult mouse and human β-cell proliferation via inhibition of the mTOR pathway.^174^ Another example is miR-184, which targets Argonaute2, a component of the miRNA-induced silencing complex, to prevent murine β-cell expansion.^175^ Silencing of miR-184 during insulin resistance promotes expression of Argonaute2, which in turn, facilitates the function of miR-375 to decrease the expression of growth suppressors and promote compensatory β-cell proliferation to meet metabolic demands.^175^

Epigenetic factors also regulate β-cell proliferation. One well-defined example has been elucidated in children with a focal form of congenital hyperinsulinism (CHI), in which focal β-cell expansion occurs at a single-specific location in the pancreas as a result of two unique events. First, a paternally inherited mutation in *ABCC8* or *KCNJ11* in chromosome region 11p15.1 leads to increased insulin secretion. Second, there is loss of the maternal 11p15.5 region, which is paternally imprinted and only expressed from the maternal allele. This region encodes the gene for p57^KIP2^, a key cell cycle inhibitor, as well as the lncRNA, *H19*, which, in turn, encodes a miRNA (miR-675) that represses expression of the gene for the IGF-1 receptor.^176,177^ This causes an imbalance in the expression of imprinted genes and the paternally expressed growth factor, IGF2, at 11p15.5, leading to β-cell hyperplasia.^178^

Another example is histone modifications through trimethylation of H3K27 or H3K4 that are regulated by Polycomb or Trithorax group protein complexes, respectively.^179,180^ In juvenile rodent and human β-cells, this restricts access to promoters of genes encoding cell cycle inhibitors, thereby permitting β-cell proliferation, but these histone modifications are reduced in adult human β-cells, thereby restricting proliferation.

The elucidation of factors that underlie the low capacity of adult human β-cell replication under normal physiological conditions, will be important for the development of future regenerative medicine approaches to treating diabetes due to β-cell deficiency.

##### Response to injury

The pancreas is not highly regenerative after injury and although acinar and endocrine cell division is increased, tissue mass is not fully restored.^181^ In type 1 diabetes, an autoimmune destruction of insulin-producing β-cells in the pancreas leads to inadequate insulin biosynthesis and secretion. This results in hyperglycemia and the need for life-long exogenous insulin replacement. Restoration of insulin production is thus a major medical challenge given the limited regenerative potential of pancreatic β-cells. Clonogenic cells have been identified in isolated pancreatic ducts from both mice and humans.^15^ Classic murine pancreatic injury models include partial β-cell ablation, partial pancreatectomy, partial duct ligation, or caerulein treatment (see review^15^). In vivo lineage tracing experiments using such injury models have shown that these clonogenic ductal cells are not pancreatic stem/progenitor cells and do not contribute to endocrine or acinar cell regeneration. Rather, they are committed to the ductal lineage (Fig. 3c). Thus, unlike the liver, the pancreas does not have bona fide stem/progenitor cells.

Under conditions of metabolic stress or pancreatic inflammation, hybrid cells co-expressing different endocrine hormones, or ductal structures with characteristics of both acinar and ductal cells, respectively, are often observed.^182,183^ Lineage tracing experiments in mice indicate that these hybrid cells arise due to transdifferentiation events.^15^ In some instances, transdifferentiation is transient and reversible once the insult is eliminated, for example in inflammation-induced acinar-to-ductal metaplasia, where acinar cells transiently convert into proliferative duct-like cells, which then re-differentiate into acinar cells to repair tissue damage.^184^ Reversible β-cell dedifferentiation is observed upon reduction of elevated blood glucose^182^ (Fig. 3c). This transient transdifferentiation response is similar to hepatocyte-to-ductal cell conversion in the liver. If the insult is severe, dedifferentiation of differentiated cells can result in reversion to a stem-like state for participation in tissue repair.^185,186^ With severe or long-lasting insults, transdifferentiation events can become permanent.^187^ Moreover, as with liver, the regenerative response of the mammalian pancreas can vary depending on the injury model.^188^

##### Conclusion

Liver and pancreas utilize mature cell types for cell regeneration under both homeostatic and injury conditions. In cases of severe injury, both liver and pancreas depend on the plasticity of differentiated cells to either revert to a stem/stem-like state or to transdifferentiate. The signals that control cell plasticity would be an important area of further investigation.

### Regeneration in tissues showing active renewal

#### Intestine

The intestine is structured to maximize nutrient absorption, while preventing infection by gut-resident microbes. It is organized into crypt-villus units; a structure found in birds and mammals, but lacking in fish, insects and hydra, which have a folded, smooth and sac-like intestinal epithelium, respectively.^189–191^

##### Tissue homeostasis

**Fig. 4:**
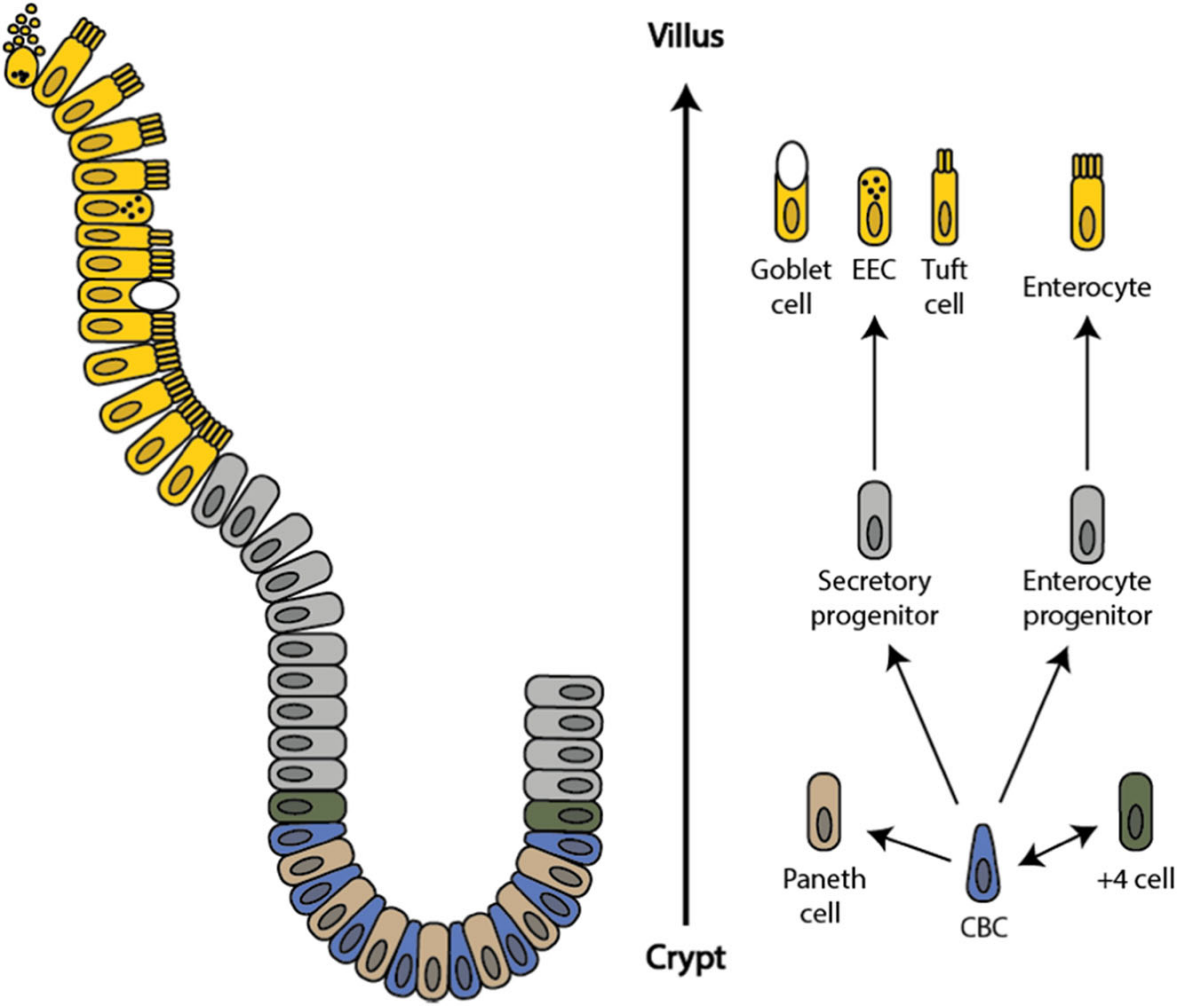
The intestinal crypt-villus unit. The intestinal crypt-villus unit is maintained by multipotent crypt base columnar (CBC; Lgr5^+^) and +4 cells (Hopx^+^, Bmi1^+^, mTert^+^, Lrig^+^). These stem cells are found at the crypt and supply the villus with specialized intestinal cells, including enterocytes, goblet cells, enteroendocrine cells (EEC), and tuft cells, which are eventually shed at the villus tip. Conversely, Paneth cells are mature cells that remain at the crypt and modulate the stem cell environment. Figure adapted, with permission from Company of Biologists, from Beumer et al.^192^

The cells of the mammalian intestinal epithelium are constantly being renewed via a conveyor belt of cells that originate at the base of the crypt and succumb by apoptosis after reaching the villus tip, 4–5 days after their generation (Fig. 4). This level of cell turnover is necessary given the mechanical, microbial and enzymatic stress to which intestinal cells are exposed.^190,192^

All intestinal epithelial cells are derived from multipotent, self-renewing, Lgr5^+^ crypt base columnar (CBC) cells.^190,192^ A second intestinal stem cell type has been identified at the +4 position of the crypt (named +4 cells; Bmi1^+^, Lrig1^+^, Hopx^+^, mTert^+^) and has the potential to replace CBCs upon injury (Fig. 4).^190,192^

Of the intestine’s differentiated cells, long-lived (~20 days) Paneth cells remain at the crypt where they secrete antimicrobial factors and modulate the stem cell environment.^192^ All other intestinal epithelial cell types become increasingly specialized as they move toward the villus tip. These mature cells include mucus-secreting goblet cells, hormone-producing enteroendocrine cells (EEC), mechanosensing tuft cells and nutrient-absorbing enterocytes.^192^

A number of key interrelated pathways have been identified in maintaining the intestine’s resident stem cell and crypt-villus structure. The crypt environment is high in Wnt-, Notch-, and EGF-, while being low in Hippo- and BMP-signaling, and this is largely regulated by the crypt’s Paneth cell population.^192,193^ Canonical Wnt signaling is initiated by Wnt3 secreted by Paneth cells or Wnt2b secreted by mesenchymal cells, which bind Frizzled7 and are necessary for crypt formation and maintenance of the Lgr5^+^ CBC population.^192–196^ Binding of secreted Wnt agonists, R-spondin 1–4, to Lgr4/5/6 on CBCs further enhances Wnt signaling and maintenance of the stem cell population.^192,194,196,197^ Active Wnt signaling causes nuclear localization of the Hippo effector, Yap, at the base of the crypt, which acts to promote CBC proliferation. When Wnt is inactive (at the villus), Yap remains cytoplasmic and is unable to exert its pro-proliferative effects.^198^ BMP forms an inverse gradient to Wnt, with low levels at the crypt and high levels at the villus, and suppresses Wnt signaling, thus promoting villus cell differentiation and preventing the formation of ectopic crypts.^199,200^

Paneth cells also produce EGF, which activates EGF receptors on the surface of CBCs and is important for cell proliferation and maintenance of the crypt.^193^ They also produce Delta-like ligand 1 and 4 (Dll1 and Dll4), activating the Notch receptor on the surface of CBCs and preventing their differentiation into goblet cells.^193^ Dll1 is also produced by secretory progenitors to promote the differentiation of neighboring cells into enterocytes, thus contributing to the organization and diversity of the villus.^201^

The structure and high turnover of the intestinal epithelium allows a rapid response to environmental factors. In rodents, the dynamic nature of the intestine has been demonstrated with prolonged fasting, resulting in a reduction in villus length and number.^202,203^ This tissue plasticity is also responsible for the intestine’s high-regenerative capacity in response to a range of physical and environmental insults.

##### Response to injury

Acute intestinal damage, due to inflammation, irradiation or infarction, can lead to relatively rapid, complete regeneration.^204–206^ However, completeness of the regenerative response varies depending on the type of injury, with repair being incomplete with chronic injury or full thickness wounds. Some models of chemically induced colitis can be used to elicit a chronic inflammatory injury with severe ulceration and loss-of-crypts.^206^ In addition, focal biopsy injury can be used to introduce full thickness wounds from which repair is rapid but new crypts are irregularly formed.^196^ Furthermore, the regenerative response can be delayed with aging.^207^

Regardless of the mechanism of injury, the regenerative process follows a similar sequence of events. Following the initial reestablishment of the intestinal barrier and tissue debridement, crypt hyper-proliferation and fission restores structure and function, a process highly dependent on resident stem cells.^208^ Interestingly, even with a depletion of CBCs as a result of radiation injury, intestinal repair and homeostasis is retained, suggesting involvement of an alternative stem-cell source or a gain of stem cell characteristics by non-stem cells.^209^ Lineage tracing studies have demonstrated the multi-potentiality of the +4 reserve stem cell.^210,211^ Furthermore, loss-of-CBCs promotes dedifferentiation of secretory and enterocyte progenitors and a recovery of their stem cell potentials.^201,212^

With injury, cells and tissues outside of the crypt-villus unit are also involved in regulating the repair response. These include mesenchymal cells, fibroblasts, immune cells, enteric neurons and capillaries. Both resident and recruited macrophages play an important role in intestinal repair. In the gut, macrophages cannot strictly be categorized as M1 or M2 as they express markers of both subtypes.^213^ However, they do play many of the roles expected of a M1 or M2 macrophage. Their immediate task is to promote the clearance of microbes, and following this inflammatory phase, they are necessary for the recruitment of mesenchymal cells and fibroblasts for tissue repair.^214^

Mesenchymal cells respond to endothelial cell signals and control the innate and adaptive immune response in the connective tissue layer.^215^ In addition, they regulate fibroblast proliferation, type I collagen deposition and myofibroblast differentiation to avoid fibrosis.^56,192^ Mesenchymal cells are also key to the repair response, producing prostaglandin E_2_ to promote the differentiation of wound-associated epithelial cells early in the repair response, and prostaglandin I_2_ to promote angiogenesis.^196^

A number of signaling pathways are required for intestinal regeneration, with Wnt (via Wnt5 non-canonical ligand), Yap and EGF signaling being activated during the regenerative process.^192,196,208^ Wnt5, produced by mesenchymal cells surrounding the wound bed, is necessary for crypt regeneration in the mammalian intestine.^196,208^ This is consistent with tissue regeneration observed in highly regenerative species, such as zebrafish and hydra, which are also dependent on non-canonical Wnt signaling.^216,217^ However, the regenerative role of Wnt5 differs from development, where Wnt5 regulates the proximal-distal axis of outgrowing structures, such as limbs and the intestinal tract, but is not required for patterning of the crypt-villus unit.^218,219^ In the regenerating mammalian intestine, negative-feedback from Yap keeps Wnt signaling in check and controls the regenerative process.^192^

##### Conclusion

While the intestine is one of the most regenerative mammalian tissues, this regenerative process is dependent on the type and severity of the injury. As suggested by Miyoshi, ‘wound healing prioritizes rapid functional recovery rather than structural integrity’.^196^ The ultimate goal is restoration of a protective barrier to luminal microbes and recovery of efficient nutrient absorption. Effective intestinal regeneration is achieved through control of the initial inflammatory phase and prevention of an overactive fibrotic response.

#### Skin and hair follicle

**Fig. 5:**
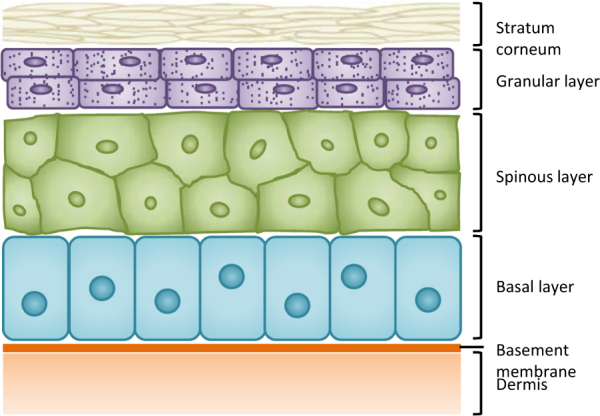
The interfollicular epidermis. The interfollicular epidermis is stratified into four layers: the basal, spinous, and granular layers and stratum corneum. Basal progenitor cells differentiate as they lose contact with the basement membrane and migrate toward the skin’s surface where they are eventually shed. Figure adapted, with permission from Springer Nature, from Hsu et al.^221^

The skin has an important barrier function that prevents dehydration and microbial invasion, and aids thermoregulation. It is made up of three layers, the epidermis, dermis and subcutaneous fat, and contains a number of appendages, including hair follicles, glands, and nails (Fig. 5).

##### Tissue homeostasis

The interfollicular epidermis is stratified into four layers (from inner to outer: basal membrane, spinous layer, granular layer, and stratum corneum) that are maintained via a columnar movement and simultaneous differentiation of basal cells from the basement membrane to the skin’s surface. Basal progenitors are found in the innermost layer, the basal layer, where they express integrins for adherence to the basement membrane and keratins, K5 and K14, for mechanical resistance (Fig. 5).^220,221^ Most basal cell division is asymmetric, occurring every 6–7 days, pushing cells further away from the basement membrane cues required to maintain their stem cell state.^222^ As basal cells differentiate into keratinocytes they move into the spinous layer and begin to express K1 and K10 keratins. They are then pushed into the granular layer and finally into the dead stratum corneum, before being shed. The entire process, from basal progenitor to shedding, takes 2–3 weeks in the mouse and ~4 weeks in the much thicker human epidermis.^220,222^

**Fig. 6:**
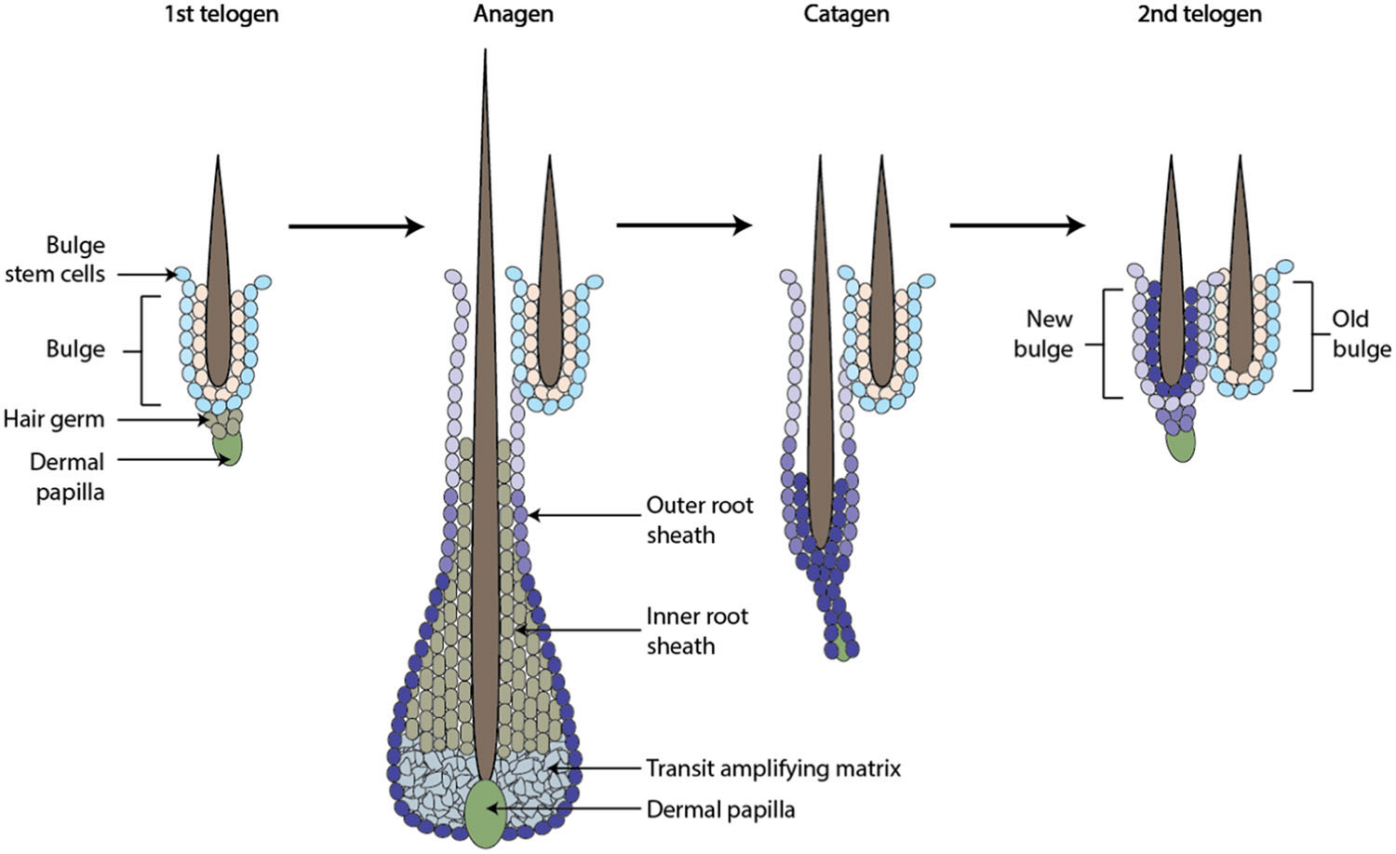
The hair follicle. The hair follicle cycles through three phases: anagen (growth), catagen (regression) and telogen (rest). Bulge stem cells supply the outer root sheath, while hair germ cells at the dermal papilla generate the hair shaft and inner root sheath. During catagen, the inner root sheath and much of the outer root sheath regresses. However, some of the upper, middle, and lower cells of the outer root sheath generate a new bulge adjacent to the old bulge, contributing to the outer bulge, hair germ, and inner bulge, respectively. Figure adapted, with permission from Springer Nature, from Hsu et al.^221^

Hair follicles span the dermis and epidermis, cycling through three stages: anagen (growth phase), catagen (regression), and telogen (resting phase) (Fig. 6).^221^ Regeneration of the hair follicle after telogen requires bulge stem cells to generate the outer root sheath, while hair germ cells from the dermal papilla generate the hair shaft and inner root sheath.^221^ Bulge stem cells are Lgr5^+^ and largely quiescent, but undergo one to three cell divisions once in the hair germ.^222^ Bulge stem cells are multipotent and capable of differentiating into all cells of all epidermal lineages: interfollicular epidermis, hair follicle and sebaceous gland.^222^

Homeostasis of sebaceous glands does not normally involve bulge stem cells, but rather a pool of bipotent sebaceous progenitor cells (MTS24^+^, Lrig1^+^, Blimp1^+^, Lgr6^+^).^222^ These progenitors are mostly involved in maintaining the gland, but can also contribute to the interfollicular epidermis.^222^ The fourth and final dermal stem cell is the isthmus-resident stem cell (MTS24^+^, Lgr1^+^, Lgr6^+^), which is also involved in sebaceous gland homeostasis, but can be stimulated to differentiate into all dermal lineages in vivo.^222,223^

Key signaling pathways involved in the maintenance of the skin’s stem cell populations, include Wnt, Hippo, Notch, Shh, and BMP. Yap localizes to the nucleus in basal stem cells and promotes proliferation.^222,224^ Wnt signaling has also been shown to promote the proliferation of keratinocytes in the interfollicular epidermis.^225^ In the dermal papilla, Wnt/β-catenin signaling is involved in both anagen and de novo hair follicle formation.^226,227^ Shh, which is downstream of Wnt, is expressed by all stem cells of the hair follicle and sebaceous gland and is required for cell expansion and differentiation.^222^ Given this role, Shh is expressed at specific stages of the hair follicle cycle, and as with Wnt, ectopic Shh prolongs anagen.^222,227^ Wnt and Shh signaling is inhibited by Notch1-4, which bind Delta-like 1, Jagged1, and Jagged2 on surrounding target cells.^222,228^ This allows differentiation of cells of the hair follicle, interfollicular epidermis, and sebaceous gland. Other negative regulators of stem cell proliferation also control skin homeostasis. BMP2 and BMP4, produced by dermal fibroblasts and adipocytes, suppress proliferation and are necessary for the quiescent phase of bulge stem cells.^229^ Furthermore, quiescent bulge stem cells produce BMP6, keeping them in a dormant state.^222^

##### Response to injury

Skin repair has been extensively studied using an array of injury models, including incision, excision, ischemia, or burn injury.^222,230–232^ While some lower vertebrates can achieve complete epimorphic regeneration of skin after wounding, in most mammals wound healing results in scar formation, which restores the skin barrier, but lacks appendages, such as hair follicles and sweat glands that are required for normal skin function.^233^

While not always regenerative, healing of incisional and excisional wounds is effective in rodents and occurs by re-epithelialization and contraction of the wound.^234–236^ As contraction does not usually occur in human wounds, splinting of rodent wounds is often implemented to more closely model human healing outcomes and results in healing through granulation, re-epithelialization, cell proliferation, and angiogenesis.^235–238^ With burn injuries, the extent of re-epithelialisation is dependent on the depth of the wound, which can be controlled by adjusting the temperature applied.^231^

The stem/progenitor cells of the epidermis and associated appendages are important for restoring a functional dermal barrier. Skin wound healing begins with the expansion of keratinocytes at the wound’s edge, mediated by an increase in asymmetric stem cell proliferation.^239^ In addition to basal progenitor cells that are recruited to the wound area, stem cells of the isthmus-region and multipotent bulge stem cells also migrate to the wound after injury, where they are involved in re-establishing the epithelial barrier.^223,240–244^

The regenerative capacity of fetal skin is far greater than that of adults, perhaps due to a dampened immune response and highly dynamic ECM response in the former, with high levels of hyaluronic acid and the prevalence of collagen type III over collagen type I (refs. ^245–247^). In the fetus, both incisions and full thickness excisions regenerate fully, complete with the regeneration of hair follicles and glands.^245,246,248,249^ This process is dependent on tissue innervation.^31^ However, the same is not true for burn injuries, which are necrotic wounds that result in excessive macrophage recruitment and scar formation.^245,250^

The best example of mammalian epimorphic skin regeneration is in the African spiny mouse. Compared with *Mus musculus*, spiny mouse wounds re-epithelialise rapidly and regenerate dermis and epidermis, complete with hair follicles, after full thickness skin excisions.^63,64^ Similar to fetal skin wound healing, that of the spiny mouse involves an altered inflammatory response, reduced myofibroblast recruitment, high levels of ECM synthesis/turnover, and is composed predominantly of type III, rather than type I collagen.^63,64^ Interestingly, spiny mouse skin also has a 20-fold lower tensile strength compared with *Mus musculus*, consistent with the former’s autotomous skin shedding followed by rapid healing that allows predatory escape.^64^

##### Conclusion

The skin’s many appendages and stem cell/progenitor populations add complexity to its homeostasis and wound healing. As with intestinal repair, skin healing is dependent on the type of wound, with regeneration being incomplete following full thickness, necrotic, or chronic injuries. Lessons from fetal models and highly regenerative species demonstrate that control of inflammatory responses, rapid ECM remodeling and the deposition of collagen type III are necessary for complete scarless regeneration, suggesting a line of investigation for the development of therapies to improve skin regeneration.

## Conclusions and future perspectives

It is clear from the above considerations that regenerative mechanisms and the extent of regeneration in different tissues vary widely. However, some common themes are evident. Modification of inflammatory processes and the resulting inhibitory extracellular matrices following trauma are likely to be important for improving clinical outcomes in all tissues. Regeneration is favored if the inflammatory response following injury is short-lived, and if the injury is acute and not chronic. Macrophages and Tregs have an essential role in guiding the regenerative process; however, in the CNS, steroid treatment to dampen the inflammatory response is not sufficient to provide reproducible and meaningful clinical recovery in patients with spinal cord or brain injury. Further study is required to determine how immune modulation can improve wound healing in individual tissues.

Regeneration is also favored if the ECM is not rigid and if injury is not severe enough to induce a fibrotic response. At least in skin, scarless regeneration is only observed if the ratio of collagen III to I is high. An important area of future research will be to generate favorable ECM remodeling conditions post-injury that will modify cell phenotype and function to allow tissue regeneration without dysfunction. Studying the positive and negative-feedback loops between cells and the ECM during physiological and pathological remodeling will advance our understanding and help identify targets for intervention to develop regenerative and anti-fibrotic therapies.

A series of important pathways appear to control the maintenance and proliferation of tissue resident stem/progenitor cells, whether in tissues with high or low turnover, such as the intestine or brain, respectively. These include Hippo, Wnt Notch, Shh, EGF, and BMP. Manipulation of these interrelated pathways in poorly regenerative tissues may be a fruitful avenue for regenerative medicine.

In hepatocytes, β-cells and CMs, which show little or no cell turnover, insults, particularly if severe, render mature differentiated cells plastic, with mature cells reverting to a stem-like state or transdifferentiating. There is clearly a need to identify signals that control cell plasticity as this may help the development of therapeutic strategies for tissue repair. Hepatocytes and CMs also undergo endoreduplication, which leads to polyploidy and/or polynucleation that increases with age. This phenomenon is absent from resident stem cells of the skin and intestine that are being renewed continuously. Further work is required to understand the significance of polyploidy and polynucleation on the life cycle of these cells.

Determining the impact of interventions to enhance regeneration requires definition of a standard set of parameters that can be measured. A reasonable gold standard would be scarless regeneration, as observed, for example, for fetal skin and liver.^157,246^ An alternative gold standard would be scarless repair with complete restoration of structure and function, as observed after myocardial injury to the heart in adult zebrafish, or in embryonic, P1 or P2 murine hearts.^23,102,109^ However, interventions reported to date rarely result in such complete reparative regeneration in adult mammals. Given that several mitogenic pathways implicated in regenerative responses might impact equally on cell survival, reductions in scar volume observed with these agents could be due to protective effects on tissue rather than to regeneration. Immunohistochemical evidence using BrdU/EdU labeling and mitotic/cytokinetic markers may under or overestimate the amount of proliferation. DNA synthesis or S-phase marking dyes are usually given daily and are readily excreted, only allowing a short time to mark new DNA synthesis. Furthermore, DNA intercalating dyes, such as BrdU/EdU, may stress the cells by initiating damage response pathways. Adverse effects from these stains are well known in the area of neurobiology.^251^ Additionally, it is difficult to determine whether these cells will ultimately divide versus become polyploid or polynuclear. Furthermore, mitosis/cytokinesis markers, such as phospho-histone 3 (pH3) and Aurora kinase B (that also phosphorylates histone 3), provide only a snapshot of cells in the cell cycle and are likely to under estimate proliferation, especially with in vivo studies where cells are not synchronized. These strategies, which involve extrapolation from samples restricted to small areas of the tissue, are predicated on cell proliferation being homogeneous throughout the tissue. However, cells may respond differently to mitogenic stimuli depending upon the regional location of these cells within the tissue. Therefore, based on these considerations investigators reporting regeneration in their studies should optimally determine the following parameters: total cell numbers in a tissue, cell survival/apoptosis, cell proliferation, total scar volume, and scar volume as a percent of whole tissue. Availability of these parameters allows quantification of the replacement of scar tissue by new cells and, thus, the extent of the regenerative response after injury.

### Data availability

The data sharing is not applicable to this article as no data sets were generated.
